# Identification of pharmacogenetic variants from large scale next generation sequencing data in the Saudi population

**DOI:** 10.1371/journal.pone.0263137

**Published:** 2022-01-28

**Authors:** Ewa Goljan, Mohammed Abouelhoda, Mohamed M. ElKalioby, Amjad Jabaan, Nada Alghithi, Brian F. Meyer, Dorota Monies

**Affiliations:** 1 Clinical Genomics, Centre for Genomic Medicine, King Faisal Specialist Hospital and Research Centre, Riyadh, Saudi Arabia; 2 Saudi Human Genome Program, King Abdulaziz City for Science and Technology, Riyadh, Saudi Arabia; 3 Computational Biosciences, Centre for Genomic Medicine, King Faisal Specialist Hospital and Research Centre, Riyadh, Saudi Arabia; Texas Tech University Health Science, Lubbock, UNITED STATES

## Abstract

It is well documented that drug responses are related to Absorption, Distribution, Metabolism, and Excretion (ADME) characteristics of individual patients. Several studies have identified genetic variability in pharmacogenes, that are either directly responsible for or are associated with ADME, giving rise to individualized treatments. Our objective was to provide a comprehensive overview of pharmacogenetic variation in the Saudi population. We mined next generation sequencing (NGS) data from 11,889 unrelated Saudi nationals, to determine the presence and frequencies of known functional SNP variants in 8 clinically relevant pharmacogenes (*CYP2C9*, *CYP2C19*, *CYP3A5*, *CYP4F2*, *VKORC1*, *DPYD*, *TPMT* and *NUDT15*), recommended by the Clinical Pharmacogenetics Implementation Consortium (CPIC), and collectively identified 82 such star alleles. Functionally significant pharmacogenetic variants were prevalent especially in CYP genes (excluding *CYP3A5*), with 10–44.4% of variants predicted to be inactive or to have decreased activity. In *CYP3A5*, inactive alleles (87.5%) were the most common. Only 1.8%, 0.7% and 0.7% of *NUDT15*, *TPMT* and *DPYD* variants respectively, were predicted to affect gene activity. In contrast, *VKORC1* was found functionally, to be highly polymorphic with 53.7% of Saudi individuals harboring variants predicted to result in decreased activity and 31.3% having variants leading to increased metabolic activity. Furthermore, among the 8 pharmacogenes studied, we detected six rare variants with an aggregated frequency of 1.1%, that among several other ethnicities, were uniquely found in Saudi population. Similarly, within our cohort, the 8 pharmacogenes yielded forty-six novel variants predicted to be deleterious. Based upon our findings, 99.2% of individuals from the Saudi population carry at least one actionable pharmacogenetic variant.

## Introduction

Pharmacogenomics (PGx) studies genetic variations in an individual’s drug metabolizing enzymes, associating these with adverse drug events or the level of drug response [1]. Drug efficacy and toxicity may be predicted from the genetic background of individuals, particularly in respect of the Cytochrome P450 (CYP) family of liver enzymes [2, 3]. These enzymes catalyze the conversion of substances that are metabolized by our bodies, including pharmaceuticals [4]. Overall, the efficacy of a drug is related to its Absorption, Distribution, Metabolism and Excretion (ADME) [3, 5]. The efficacy of a drug may also be associated with drug target polymorphisms. Drug targets can include receptors, enzymes and membrane transporters [2]. CYPs are responsible for deactivation of many drugs through direct metabolic activity or via facilitation of excretion and thus play a central role in ADME related efficacy. CYPs are also important in enzymatic conversion of some drugs from their native to bioactive forms [6].

These differences in drug metabolism highlight the current trend towards individualized pharmacotherapy, such that the right drug is delivered at the right dose to the right patient. A standard dose of a given drug is not always safe, effective or economical in an individual patient [7, 8]. The high incidence of adverse drug events (ADEs) represents a heavy burden for the US health care system. Almost 7 million emergency department visits are related to ADEs each year with an estimated cost of $3.5 billion annually [9]. Large scale genomic studies provide opportunities to associate drug responses with individual pharmacogenetic profiles. Such knowledge may improve drug efficacy, result in better outcomes, and in some instances, prevents life-threatening adverse drug events. Dosing no longer needs to be based on the average drug responses of a patient population but can be personalized, taking into consideration individual pharmacogenomic and environmental variation. There are well-established drug-gene interactions, that include but are not limited to, clopidogrel (*CYP2C19*), warfarin (*CYP2C9*, *VKORC1* and *CYP4F2*), thiopurines (*TPMT*, *NUDT15*), tacrolimus (*CYP3A5*) and fluorouracil (*DPYD*) [10–14]. These medications are commonly used globally with Saudi Arabia being no exception [9, 15]. Protocols targeting use of the right drug at the right dose in the right person, based on genomic data to personalize treatment, have already been clinically implemented successfully in other countries, e.g. the RIGHT protocol in the US and U-PGx project in Austria, Spain, Great Britain, Greece, Italy, Netherlands and Slovenia [16–18]. The allelic frequencies of genes encoding drug-metabolizing enzymes and their phenotypic consequences may vary considerably between ethnic groups. The impact of these allelic variants has been well studied in Caucasians and some other ethnicities, yet poorly in Arabs [5, 19]. This study expands pharmacogenetic knowledge of the Arab population. Description of the allelic spectrum of pharmacogenes, both known and novel variants, their frequency, and phenotypic designation in Saudi nationals, will provide a basis for better clinical management in this population.

During the last decade technological advances have enabled comprehensive mapping of human pharmacogenes [20, 21]. Next Generation Sequencing (NGS) and High Performance Computing (HPC) are two technologies that have enhanced this field [22, 23]. The mining of variants using sequence data from population-based genome programs, provide an opportunity to characterize the pharmacogenomic profiles of each of these groups. Here we describe our findings from the Saudi population.

## Results

Mining of NGS data from a total of 11,889 (1,928 PGx gene panels and of 9,961 exomes) unrelated individuals was used to impute allele and haplotype frequencies. We analyzed frequencies of 82 haplotypes distributed across 8 pharmacogenes (Table 1). Nineteen *CYP2C9* variants (*2, *3, *5, *6, *7, *8, *9, *11, *12, *14, *24, *32, *33, *36, *39, *43, *44, *45, *60) were identified that jointly accounted for 21.1% of all *CYP2C9* alleles in Saudi Arabs, however, only CYP2C9*2 with a minor allele frequency (MAF) of 13.4% and CYP2C9*3 (MAF = 5.3%) were relatively common. Fifteen variant alleles (*2, *3, *4A, *6, *8, *9, *12, *13, *15, *16, *17, *24, *28, *30, *34) were found in *CYP2C19* of which *17 and *2 were the most common: 25.9% and 9.6%, respectively. A splice site variant (rs7767746) that is the core allele for CYP3A5*3 was present in 84.7% of the population. Three other alleles (*6, *7 and *8) in *CYP3A5* showed MAFs from <0.1% to 2.4%. In *CYP4F2*, 44.4% of Saudi individuals harbor a *3 allele, the remaining population being wild type. We detected four *VKORC1* alleles; the most common was VKORC1*2 (MAF = 53.7%) followed by rs7294, 3730G>A (MAF = 29.2%). Two other *VKORC1* variants: 106G>T (rs61742245) and 196G>A (rs72547529) were less commonly observed with MAFs of 2.1% and <0.1%, respectively. Genetic polymorphisms in *DPYD* and *TPMT* were rare in the Saudi population. We identified eight variants (rs67376798, rs3918290, rs1801266, rs115232898, rs112766203.1, rs72549304, rs146356975, rs56038477) for *DPYD* and ten star alleles for *TPMT* although the overall MAFs for both these were low, 0.7% (*DPYD*) and 0.9% (*TPMT*). Two alleles (*3 and *5) were identified in *NUDT15*. The *3 allele was present with a MAF of 1.8%; *5 being much less common (MAF<0.1%), in the population.

**Table 1:** Frequencies and functional status of pharmacogenetic alleles in Saudi population.

Gene	Allele	Core variant	Variant type	Functional Status	Allele Frequency, SA (%)
***CYP2C9*** *					
	*1	None		Normal	78.9
	*2	rs1799853	Missense (R144C)	Decreased	13.4
	*3	rs1057910	Missense (I359L)	Inactive	5.3
	*4	rs56165452	Missense (I359T)	Decreased	0
	*5	rs28371686	Missense (D360E)	Decreased	0.2
	*6	rs9332131	Frameshift	Inactive	0.1
	*7	rs67807361	Missense (L19I)	Uncertain function	<0.1
	*8	rs7900194	Missense (R150H)	Decreased	0.5
	*9	rs2256871	Missense (H251R)	Normal	0.7
	*11	rs28371685	Missense (R335W)	Decreased	0.6
	*12	rs9332239	Missense (P489S)	Decreased	<0.1
	*13	rs72558187	Missense (L90P)	Inactive	0
	*14	rs72558189	Missense (R125H)	Decreased	<0.1
	*24	rs749060448	Missense (E354K)	Inactive	<0.1
	*32	rs868182778	Missense (V490F)	Uncertain function	<0.1
	*33	rs200183364	Missense (R132Q)	Inactive	0.3
	*36	rs114071557	Start lost	Uncertain function	<0.1
	*39	rs762239445	Missense(R124W)	Inactive	<0.1
	*43	rs767576260	Missense (R124W)	Inactive	<0.1
	*44	rs200965026	Missense (T130M))	Decreased	<0.1
	*45	rs199523631	Missense (R132W)	Inactive	<0.1
	*60	rs767284820	Missense (L467P)	Uncertain function	<0.1
***CYP2C19*** *					
	*1	None		Normal	63.3
	*2	rs4244285	Splicing defect	Inactive	9.6
	*3	rs4986893	Stop-gain (W212X)	Inactive	0.1
	*4A	rs28399504	Start lost	Inactive	<0.1
	*4B	rs28399504, rs12248560	Start lost, Regulatory	Inactive	0
	*5	rs56337013	Missense (R433W)	Inactive	0
	*6	rs72552267	Missense (R132Q)	Inactive	<0.1
	*7	rs72558186	Splicing defect	Inactive	0
	*8	rs41291556	Missense (W120R)	Inactive	0.1
	*9	rs17884712	Missense (R144H)	Decreased	0.2
	*10	rs6413438	Missense (P227L)	Decreased	0
	*12	rs55640102	Stop-lost (X491C)	Uncertain function	<0.1
	*13	rs17879685	Missense (R410C)	Normal	0.4
	*15	rs17882687	Missense (I19L)	Normal	0.4
	*16	rs192154563	Missense (R442C)	Decreased	<0.1
	*17	rs12248560	Regulatory	Increased	25.9
	*24	rs118203757	Missense (R335Q)	Inactive	<0.1
	*28	rs113934938	Missense (V374I)	Normal	<0.1
	*30	rs145328984	Missense (R73C)	Uncertain function	0.1
	*34	rs367543002, rs367543003	Missense (P3S, F4L)	Uncertain function	<0.1
***CYP3A5*** *					
	*1	None		Normal	12.5
	*2	rs28365083	Missense (T398N)	Uncertain function	0
	*3	rs776746	Splicing defect	Inactive	84.5
	*6	rs10264272	Splicing defect	Inactive	2.4
	*7	rs41303343	Frameshift	Inactive	0.4
	*8	rs55817950	Missense (R28C)	Uncertain function	<0.1
***CYP4F2*** *					
	*1	None		Normal	55.6
	*3	rs2108622	Missense (V433M)	Decreased function	44.4
***VKORC1*** *					
	Wild-type	None		Normal	15.08
	1173C>T (*2)	rs9934438	Regulatory	Decreased expression	53.7
	3730G>A	rs7294	UTR	Increased	29.2
	85G>T	rs104894539	Missense(V29L)	Increased	0
	106G>T	rs61742245	Missense (D36Y)	Increased	2.1
	172A>G	rs104894541	Missense (R58G)	Increased	0
	196G>A	rs72547529	Missense (V66M)	Increased	<0.1
	292C>G	rs72547528	Missense (R98W)	Increased	0
	383T>G	rs104894542	Missense (L128R)	Increased	0
***DPYD*** * ^***/***^ **					
	*1	None		Normal	99.82
	2846A>T	rs67376798	Missense (D949V)	Inactive	<0.1
	*2A	rs3918290	Splicing defect	Inactive	0.1
	*8	rs1801266	Missense(R235W)	Inactive	<0.1
	557A>G	rs115232898	Missense (Y186C)	Decreased function	0.1
	2279C>T	rs112766203.1	Missense (T760I)	Decreased function	<0.1
	1475C>T	rs72549304	Missense (S492L)	Inactive	<0.1
	868A>G	rs146356975	Missense (K290E)	Decreased function	<0.1
	1236G>A (HapB3)	rs56038477	Synonymous (E412 =)	Decreased function	0.5
***TPMT*** **					
	*1	None		Normal	99.1
	*2	rs1800462	Missense (A80P)	Inactive	<0.1
	*3A	rs1800460, rs1142345	Missense (A154T, Y240C)	Inactive	0.3
	*3B	rs1800460	Missense (A154T)	Inactive	<0.1
	*3C	rs1142345	Missense (Y240C)	Inactive	0.4
	*6	rs75543815	Missense (Y180F)	Uncertain function	0
	*8	rs56161402	Missense (R215H)	Uncertain function	0.2
	*12	rs200220210	Missense (S125L)	Uncertain function	<0.1
	*24	rs6921269	Missense (Q179H)	Uncertain function	0.1
	*25	rs377085266	Missense(C212R)	Uncertain function	<0.1
	*34	rs111901354	Missense (R82W)	Uncertain function	<0.1
***NUDT15*** *					
	*1	None		Normal	98.5
	*3	rs116855232	Missense (R139C)	Inactive	1.8
	*5	rs186364861	Missense (V18I)	Uncertain function	<0.1

Functional status of star alleles was defined according to:

* the Pharmacogene Variation Consortium (https://www.pharmvar.org) and the Clinical Pharmacogenetics Implementation Consortium guidelines (https://cpicpgx.org/guidlines/).

**-literature [24].

**Fig 1:**
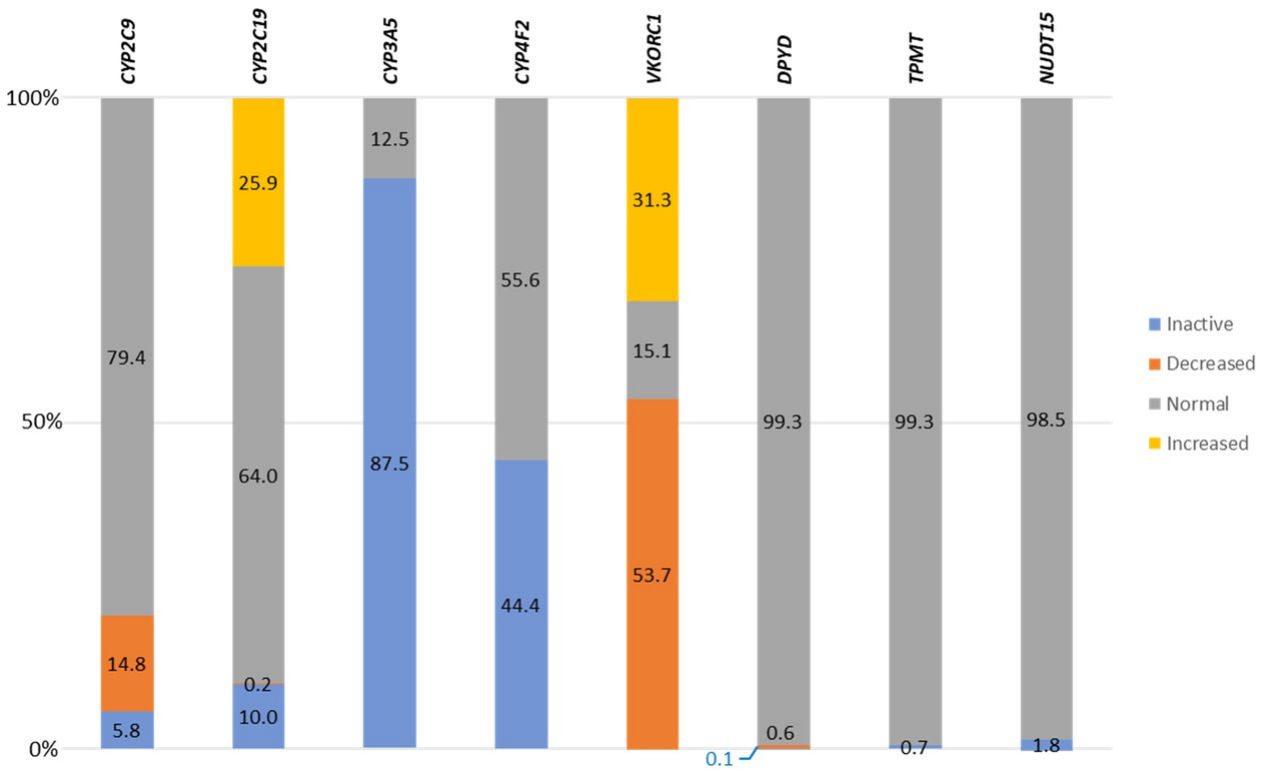
Combined functional consequences of genetic variations in pharmacogenes, within the Saudi population.

Functional consequences predicted for PGx alleles in the Saudi population were found predominantly in CYP genes. In *CYP3A5* we found the highest number (87.5%) of inactive alleles as a result of the frequently observed intronic splice site CYP3A5 *3 variant. *CYP4F2* showed decreased function alleles in 44.4% of individuals, whereas, in two other CYP genes (*CYP2C9* and *CYP2C19*) reduced function alleles (inactive or decreased) were less common, being 20.6% and 10.1%, respectively. In other prominent PGx genes, allele function was much more conserved, with only 1.8%, 0.7% and 0.7% of *NUDT15*, *TMPT* and *DPYD* variants predicted to affect activity, respectively. In contrast, functionally, *VKORC1* was highly polymorphic with 53.7% of Saudi individuals harboring variants predicted to result in decreased activity, whereas 31.3% carry variants leading to increased metabolic activity (Table 1 and S1 Table, Fig 1).

**Fig 2:**
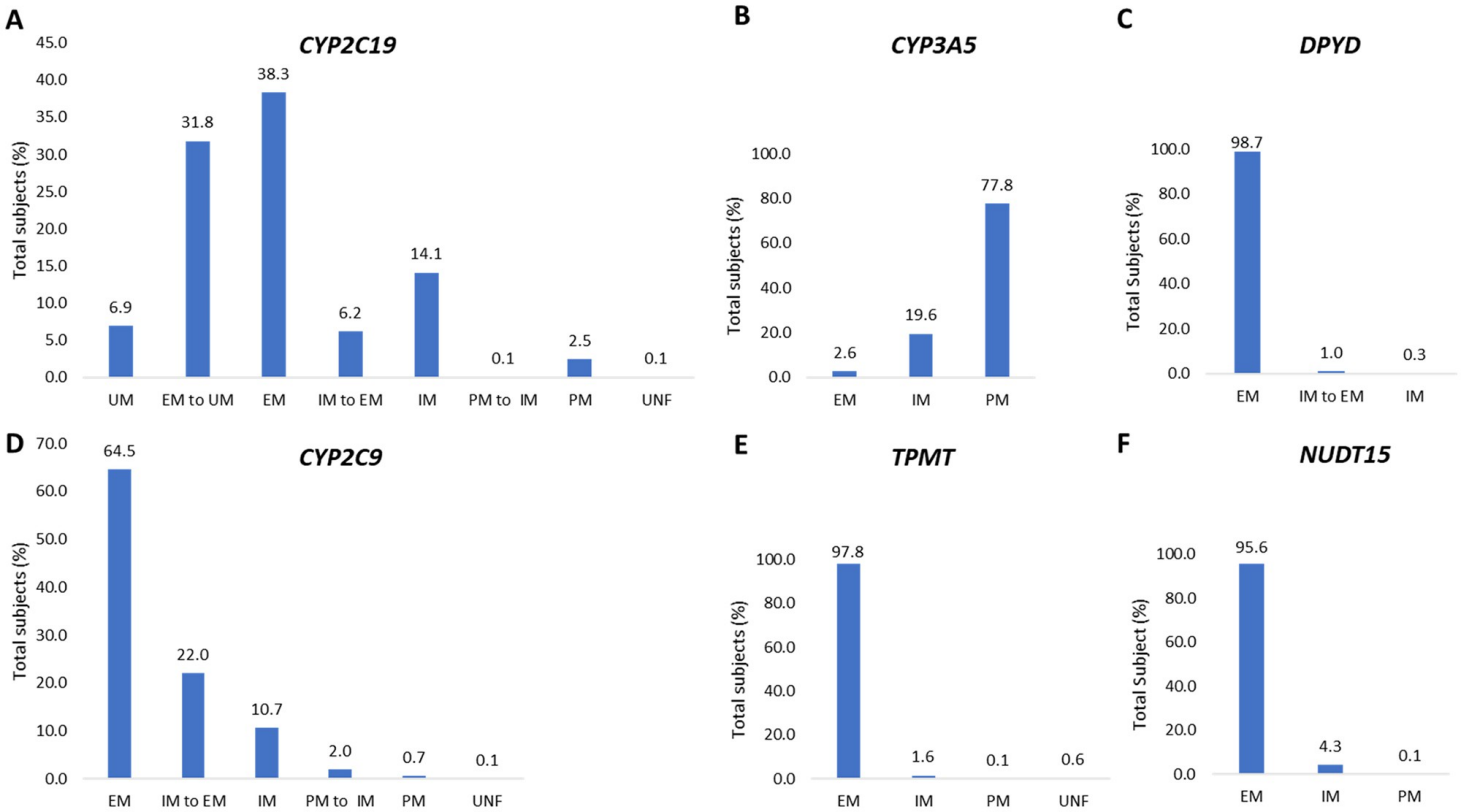
Percentage of the predicted *CYP2C19* (A), *CYP3A5* (B), *DPYD* (C), *CYP2C9* (D), *TPMT* (E), *NUDT15* (F) metabolizer groups. EM, extensive metabolizer; IM, intermediate metabolizer; PM, poor metabolizer; UM, ultrarapid metabolizer; UNF, uncertain function.

Based on genotypic data and predicted functional consequences of variant alleles we defined genotype-to-phenotype correlations (Fig 2). The phenotyping algorithms were derived from CPIC guidelines which were available only for *CYP2C19*, *CYP2C9*, *CYP3A5*, *TPMT*, *NUDT15*, and *DPYD*. Extensive metabolizer (EM) was the most frequent category for *DPYD* (98.7%), *TPMT* (97.8%) and *NUDT15* (95.6%). EM status was also the highest (64.5% and 38.3%) for *CYP2C9* and *CYP2C19* although a significant number of remaining individuals (35.4% and 61.6%) are predicted to carry an altered drug metabolizer status for these two genes. *CYP3A5* non-expressers (poor metabolizer, PM) represented 77.8% of the population.

**Fig 3:**
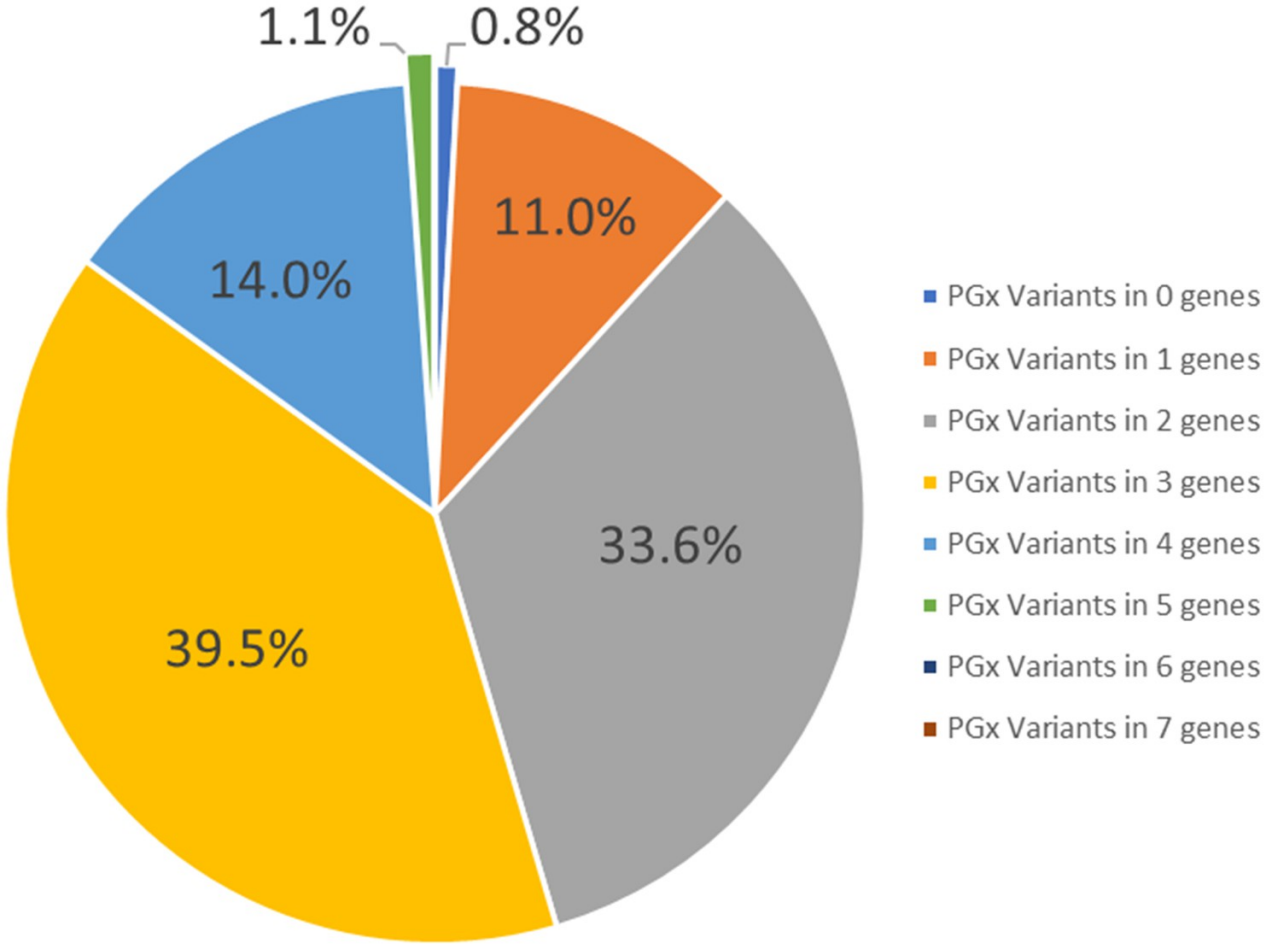
Percentage of Saudi subjects carrying actionable variants in zero to five pharmacogenomics genes.

The percentage of Saudi individuals who carry actionable PGx variant(s) is summarized in Fig 3. Of the 1928 Saudi individuals (genotyped using the PGx gene panel), 99.2% carry at least one actionable PGx allele, with a maximum of 5 detected in 1.1% of the population.

Of all 62 previously reported, predicted to be pathogenic (based on a two-fold scoring) rare variants (MAF<1%), four (1 stop-gain, 2 frameshift and 2 missense variants) with an aggregated frequency of 0.67% were uniquely observed in Saudi individuals when compared with other populations (European, Finish, Hispanic, African, South Asian, East Asian, Ashkenazi Jews and Arabs). Two missense variants were only present in Arabs (Table 2 and S2 Table). Next, we identified 46 novel sequence alterations in seven of the eight PGx genes studied. They included 5 stop-gain, 5 splice site, 1 frameshift, and 35 missense variants with an ADME score of ≥84%. *DPYD* revealed the largest number (n = 19) of novel alterations, the most frequent being *DPYD*:p.Ile971Thr, having a MAF of 0.00055 (Table 3 and S3 Table).

**Table 2:** Rare pharmacogenetic variants present in Saudi population in comparison to other populations.

			Minor allele frequency (%)										
Gene	RSID	Variant type	SA	AFR	AMR	ASJ	EAS	FIN	NFE	SAS	GME	QARB	Kaviar
***CYP2C9***	rs771127798	Frameshift	0.076	0	0	0	0	0	<0.01	0	0	0	<0.01
***CYP2C9***	rs200985348	Stop-gained	0.017	0	0	0	0	0	0	0	0	0	<0.01
***CYP3A5***	rs1267703650	Missense (I442T)	0.135	0	<0.01	0	0	0	0	0	0	0	0
***CYP4F2***	rs780094643	Missense(G417V)	0.130	0	0	0	0	0	<0.01	0	0.1	0	<0.01
***CYP4F2***	rs763539865	Frameshift	0.421	0	0	0	0	0	0	<0.01	0	0	<0.01
***DPYD***	rs568132506	Missense(P86L)	0.324	0	0.009	0	0	0	0.006	<0.01	0.05	0.3	0.007

A complete summary of rare variants in Saudi population is presented in S2 Table. SA, Saudi Arabia; AFR, Africans; AMR, Latin/Admixed Americans; ASJ, Ashkenazi Jews; EAS, East Asians; FIN, European Finish; NFE, Non-Finish European; SAS, South Asians; GME, Greater Middle East Variome; QARB, Qatari Arabs; RSID, reference SNP cluster ID.

**Table 3:** Novel pharmacogenomics variants in Saudi population.

Gene	Variant	Variant type	Minor allele frequency, SA (%)
***CYP2C19***	NM_000769.4:c.914C>A:p.Thr305Asn	missense	0.00420557
***CYP2C19***	NM_000769.4:c.332-1G>A	splice acceptor	0.00841114
***CYP2C19***	NM_000769.4:c.482-2A>G	splice acceptor	0.00841114
***CYP2C19***	NM_000769.4:c.1034T>C:p.Met345Thr	missense	0.01682227
***CYP2C19***	NM_000769.4:c.1071A>C:p.Arg357Ser	missense	0.00841114
***CYP2C9***	NM_000771.4:c.1023C>G:p.Asp341Glu	missense	0.00420557
***CYP2C9***	NM_000771.4:c.893G>C:p.Gly298Ala	missense	0.01261670
***CYP2C9***	NM_000771.4:c.961+1G>A	splice donor	0.00841114
***CYP2C9***	NM_000771.4:c.1061A>G:p.Glu354Gly	missense	0.00420557
***CYP2C9***	NM_000771.4:c.1198G>T:p.Glu400Ter	stop gained	0.01682227
***CYP2C9***	NM_000771.4:c.1243G>T:p.Glu415Ter	stop gained	0.00420557
***CYP3A5***	NM_000777.5:c.1205A>G:p.His402Arg	missense	0.00420557
***CYP3A5***	NM_000777.5:c.1120G>A:p.Glu374Lys	missense	0.00420557
***CYP3A5***	NM_000777.5:c.1067T>C:p.Leu356Pro	missense	0.00420557
***CYP3A5***	NM_000777.5:c.1063T>C:p.Tyr355His	missense	0.00420557
***CYP3A5***	NM_000777.5:c.957T>A:p.Tyr319Ter	stop gained	0.01682227
***CYP3A5***	NM_000777.5:c.931A>G:p.Ser311Gly	missense	0.00420557
***CYP3A5***	NM_000777.5:c.409T>C:p.Phe137Leu	missense	0.00420557
***CYP3A5***	NM_000777.5:c.219-2A>G	splice acceptor	0.00420557
***CYP4F2***	NM_001082.5:c.1288A>T:p.Asn430Tyr	missense	0.02102784
***CYP4F2***	NM_001082.5:c.1231G>C:p.Gly411Arg	missense	0.00420557
***CYP4F2***	NM_001082.5:c.985G>A:p.Gly329Ser	missense	0.00420557
***CYP4F2***	NM_001082.5:c.889G>T:p.Asp297Tyr	missense	0.00841114
***DPYD***	NM_000110.4:c.2836delG:p.Ala946LeufsTer2	frameshift	0.00841114
***DPYD***	NM_000110.4:c.1526C>G:p.Ser509Ter	stop gained	0.00420557
***DPYD***	NM_000110.4:c.958+1G>A	splice donor	0.00841114
***DPYD***	NM_000110.4:c.390T>A:p.Cys130Ter	stop gained	0.00420557
***DPYD***	NM_000110.4:c.2912T>C:p.Ile971Thr	missense	0.05467239
***DPYD***	NM_000110.4:c.2310C>G:p.Ile770Met	missense	0.00420557
***DPYD***	NM_000110.4:c.2137A>C:p.Asn713His	missense	0.00420557
***DPYD***	NM_000110.4:c.2083T>G:p.Cys695Gly	missense	0.00420557
***DPYD***	NM_000110.4:c.1804C>A:p.Pro602Thr	missense	0.00420557
***DPYD***	NM_000110.4:c.1679T>C:p.Ile560Thr	missense	0.00420557
***DPYD***	NM_000110.4:c.1657C>T:p.Pro553Ser	missense	0.00420557
***DPYD***	NM_000110.4:c.1591G>A:p.Val531Met	missense	0.00420557
***DPYD***	NM_000110.4:c.1405A>G:p.Met469Val	missense	0.00420557
***DPYD***	NM_000110.4:c.1309G>A:p.Ala437Thr	missense	0.00841114
***DPYD***	NM_000110.4:c.1076T>C:p.Val359Ala	missense	0.00420557
***DPYD***	NM_000110.4:c.574C>T:p.Leu192Phe	missense	0.00420557
***DPYD***	NM_000110.4:c.431C>T:p.Ala144Val	missense	0.01682227
***DPYD***	NM_000110.4:c.217C>T:p.Leu73Phe	missense	0.00420557
***DPYD***	NM_000110.4:c.194C>A:p.Thr65Lys	missense	0.00420557
***TPMT***	NM_000367.5:c.581G>A:p.Gly194Asp	missense	0.00841114
***TPMT***	NM_000367.5:c.454A>G:p.Arg152Gly	missense	0.01682227
***TPMT***	NM_000367.5:c.202C>A:p.Pro68Thr	missense	0.00420557
***VKORC1***	NM_024006.6:c.404G>A:p.Cys135Tyr	missense	0.01261670

## Discussion

Inter-individual differences in drug efficacy drive current trends towards personalized pharmacotherapy targeting delivery of the right drug, at the right dose to the right patient. A standard dose of a given drug is not always safe, effective or economical in an individual patient [7, 8]. Mining of large-scale NGS data is a very powerful tool for cataloging the range and frequency of genetic variation in populations [25]. We used whole exome and PGx gene panel NGS data to estimate pharmacogenetic diversity in the Saudi population, thus far poorly recorded in current databases, compared to many other ethnic groups.

Our analysis provides the most comprehensive overview of PGx variability (predicted to be clinically relevant), of 8 phase I and phase II enzymes, in the Saudi population, published to date. We found that 61.6% of the Saudi cohort carry actionable *CYP2C19* alleles, which may be associated with an increased risk of major adverse cardiovascular events during antiplatelet therapy with clopidogrel. In this instance ADEs range from stent thrombosis in poor and intermediate metabolizers, to bleeding risk in rapid and ultrarapid metabolizers. This drug was prescribed to several thousand patients who were treated at King Faisal Specialist Hospital and Research Centre, Riyadh, Saudi Arabia (KFSHR&RC) last year alone. Similar to European, African and Ashkenazi populations, CYP2C19*17 was the most frequent allele. CYP2C19*30 was unique to Arabs, with CYP2C19*13 and CYP2C19*15 detected in Saudi individuals, observed only in Africans (*13 and *15) and Jews (*15). Actionable *CYP2C9* alleles associated with metabolism of warfarin were identified in 35.4% of Saudis.

Furthermore, the CYP4F2*3 and VKORC1*2 variants responsible for increased [26] and decreased warfarin activity [12] respectively, were strongly represented in our study population. *CYP4F2* acts as an important counterpart to *VKORC1* in limiting excessive accumulation of vitamin K [27, 28]. Inappropriate warfarin dosing underlies one of the most frequently reported adverse events, acute haemorrhages being one of the most common emergency visits in the US [29]. At KFSH&RC alone, warfarin is prescribed for several thousand patients every year. According to updated CPIC guidelines, genotypes of *CYP2C9*, *VKORC1* and *CYP4F2*, should be considered together to estimate therapeutic warfarin dosing. One of the key factors strongly considered in dosing algorithms include ethnicity and population related genetic information. The majority of PGx data underpinning these guidelines arises from European, African American and East Asian ancestry [12]. Very little is known about pharmacogenetics in Arabs. Our study shows that the frequency of CYP2C9*2, *3, and VKORC1*2 in the Saudi population is similar to that of Europeans[25]. Other *CYP2C9* variants common in Africans and present in Europeans (e.g. CYP2C9*5, *6, *8, and *11), that should be considered in warfarin dosing algorithms due to associated bleeding risk, show low occurrence in the Saudi population. Based on our findings, and subject to clinical validation, dosing recommendations for warfarin in Saudi patients should follow those for non-African ancestry, as recommended in CPIC guidelines [22]. However, studying the impact of a significantly higher frequency, of the functionally inactive CYP2C9*33 allele, on warfarin dosing in the Saudi population is strongly indicated. The vast majority of the Saudi population carries the CYP3A5*3 variant that results in a truncated mRNA with loss of protein expression [30]. Frequency of the *3 allele varies extremely across human populations and is correlated with distance from the equator. Equatorial populations may experience shortage of water and a sodium retaining phenotype in hot climates [31]. Our findings show frequency of this allele in the Saudi population, to be similar to that in six other populations (Ashkenazi, European, American, Finish and both Asians) [24, 25]. This gene catalyzes the metabolism of tacrolimus, a mainstay immunosuppressant. Patients with the CYP3A5*3 allele require the standard dose of this medication [13]. At KFSH&RC alone ~4000 patients received tacrolimus last year, and 22.2% of these may be normal metabolizers (2.6%) or intermediate metabolizers (19.6.%), requiring an increased tacrolimus dose to achieve a successful outcome. Clinical validation of this would be required, particularly given relatedness of donors and recipients in a consanguineous population, where histoincompatibility may be less than observed elsewhere.

Genetic variation in *TPMT* and *NUDT15* are strongly linked to the risk for adverse reactions, to thiopurines commonly used for treatment of malignant and non-malignant conditions [11]. The “normal” starting doses are generally high based on clinical trials which are enriched in wild-type individuals. Full doses are tailored for normal metabolizers and may cause acute toxicity in intermediate and poor metabolizers [32]. Thiopurine tolerance is highly correlated with genetic ancestry [33]. The functionally inactive TPMT*3A allele is much less common in Saudi individuals relative to American, European and Ashkenazi populations [24, 25]. CPIC guidelines recommend a customized dose of thiopurines in compound intermediate metabolizers (intermediate metabolizers in both TMPT and NUDT15 [11]. We identified 0.03% (n = 3) compound intermediate metabolizers in Saudi population. Genetic variation in *DPYD* is a strong predictor of adverse risk related to use of the chemotherapeutic agent fluorouracil, commonly used in the treatment of various malignancies. Many cases have been reported of severe toxicities or even lethal outcome due to the DPYD poor or null metabolizer phenotype [34]. In our study we identified 1.3% of Saudi individuals who carry either a functionally normal allele plus one null or one functionally decreased allele, and would be predicted to be intermediate metabolizers. Reduced doses of fluorouracil may be indicated for these individuals [10]. More importantly our study detected in the Saudi population, the *DPYD* rare pathogenic mutation (c.257C>T) that may be responsible for severe toxicity in heterozygous patients or lethality in homozygous cancer patients treated with fluoropyrimidines [35]. We found this variant to be significantly enriched in the Saudi population with approximately 1 in every 333 individuals heterozygous for this allele. This *DPYD* allele is also present in the Qatari population (0.3%) whereas it is very rare in other populations, with frequencies (relative to the Saudi population) <36-fold in Americans, <52-fold in Europeans, <99-fold in South Asians, and was absent in other compared populations (Table 2 and S2 Table). Given the high rate of consanguinity (~60%) in Saudi Arabia, we can expect relative to outbred populations, a higher incidence of homozygotes for the *DPYD* (c.257C>T) mutation. Consanguinity increases the probability of a mate to be a carrier of the same recessive allele [36]. Thus, genotyping *DPYD* in the Saudi population may have greater clinical relevance. In most of the pharmacogenes screened we observed alleles shared with other Arabs [19, 24, 37], and some unique to the Saudi population. Amongst those shared with other Arabs, some were observed at significantly (p<0.05) different frequencies (S1 and S2 Tables).

Large-scale NGS data mining enables discovery of novel and rare pharmacogenetic alterations [3]. They are often population specific alleles and are not incorporated within current pharmacogenomic assays. Our study shows that such variants are present in the Saudi population, with computational algorithms predicting their functional significance in multiple instances. They may significantly add to knowledge of potentially actionable variants in ADME genes within the Saudi population and should be further investigated. Novel variants require experimental validation to test their functional effects in drug response [38]. Our study highlights the value of mining large NGS databases as a powerful tool, to improve knowledge of genomic variation within ADME genes, and stimulate their further investigation and eventual implementation in clinical practice. The data we present from one of the larger Middle Eastern countries, provides the most comprehensive overview of pharmacogenetic variants in Arabs, who to date are underrepresented in international genomic databases. We believe it will have both immediate and near-term clinical implications, expanding the application of pharmacogenetics and the practice of precision or individualized medicine in Arab patients.

### Study limitations

The clinical impact of variants identified by this study remain in question as information from relevant clinical trials are limited. While PGx variants are predicted to be actionable in other populations, one cannot assume that these variants will ultimately have the same impact in the Saudi population without clinical verification. Another limitation of our study is the technical constraints of exome sequencing; non-coding regions and loci with high genomic complexity are poorly, or not covered at all. Structural changes and copy number variations which may be relevant are not reliably identified by whole exome or gene panel sequencing. Thus, we were not able to call star alleles with whole gene deletions, duplications or hybrids that are common in the assignment of *CYP2D6* alleles. Accordingly, we did not include *CYP2D6* in our analysis.

Furthermore, actionable variants located in non-coding regions *CYP2C19* rs12248560, *CYP3A5* rs776746, *VKORC1* rs9934438, *VKORC1* rs7294, DPYD rs67376798 were not covered by whole exome sequencing, our data for these being exclusively obtained from the PGx custom gene panel only.

## Methods

Manuscript was based on access to fully anonymized data from Saudi Human Genome Project for which waiver of consents was granted by the IRB of King Faisal Specialist Hospital and Research Center. The dataset used for mining of pharmacogenomic variants comprised 9,961 exomes and 1,928 PGx custom gene panels (genes are listed in S4 Table), from unrelated Arab individuals sequenced by the Saudi Human Genome Program (SHGP) between 2015 and 2019, as part of a comprehensive investigation of rare diseases in the Saudi population [39, 40]. We studied eight genes for which the Clinical Pharmacogenetics Implementation Consortium (CPIC) guidelines are curated (https://cpicpgx.org/guidelines/) and present on FDA labels (https://www.fda.gov.Drugs/ScienceReseach/ucm572698). CYP star allele assignment and their clinical function was derived from Pharmacogene Variation Consortium (https://www.pharmvar.org/genes/) and CPIC allele functional tables. Metabolizer types were inferred based on CPIC guidelines and the Pharmacogenomics Knowledgebase (PharmGKB) https://www.pharmgkb.org/ and they were defined as follows: ultrarapid metabolizer (UM), intermediate metabolizer (IM), extensive/normal metabolizer (EM), poor metabolizer (PM), rapid metabolizer (RM), IM to EM and PM to EM. Our method for Star allele calling was based upon using the Stargazer algorithm (v.1.0.8). This algorithm performs statistical haplotype phasing using Beagle [41] with reference samples from the 1000 Genomes Project [42]. The Beagle method is based on localized haplotype-cluster model, which is an empirical linkage disequilibrium model that can take the local structure in the data into consideration. The Beagle algorithm is accurate and runs fast due to the use of an EM-based algorithm that literately fits the best model to the data. Afterwards, the phased haplotypes computed by Beagle are then matched to publicly available star allele information, mostly in the PharmVar database (https://www.pharmvar.org) and PharmGKB (https://www.pharmgkb.org/). Finally, Stargazer reports the star allele findings in a tabular format along with prediction of the related metabolizer information. Frequencies of intronic and UTR variants were covered only by the PGx panel and their frequency was calculated based on the cohort of 1928 individuals. Variants with MAF <1% were defined as rare and genetic alterations with frequencies that exceeded the observed frequencies in other populations (European, Finish, Hispanic, African, South Asian, East Asian, Ashkenazi Jews and Arabs) by >20-fold were considered as being “Saudi-specific”. A Chi-square test was used to determine the statistical difference for allele frequencies between different populations. A *p*-value less than 0.05 was considered significant. Next, we classified alleles as novel if they were not observed in: 1000 Genomes (phase3), gnomAD (v.3.1.1), Exac (v.0.3) and Kaviar (v.160204). Functional consequence of PGx rare Saudi-specific and novel variants was predicted using a two-fold approach. Any variants with a high IMPACT rating, such as frameshift indels or stop loss variants were considered to be deleterious [43, 44]. We then applied the ADME-optimized framework that is an ensemble of deleteriousness prediction methods for predicting deleteriousness in pharmacogenes. We used 18 prediction algorithms to compute the ADME scores including CADD, SIFT, PolyPhen, LRT (likelihood ratio test), MutationAssessor, FATHMM, FATHMM-MKL, PROVEAN, VEST3, DANN, MetSVM, MetaLR, GERP++, SiPhy, PhyloP-vertebrate, PhyloP-mammalian, PhastCons-vertebrate, and PhastCons-mammalian. ADME scores larger than 84% were considered to affect pharmacogene functionality [45]. We used phenotypes generated from Stargazer for *CYP2C19*, *CYP2C9*, *CYP3A5*, *DPYD*, *NUDT15* and *TPMT* to determine the percentage of individuals predicted to have actionable PGx variants. For *VKORC1* (rs9934438) and CYP4F2*3 (rs2108622), individuals carrying heterozygous (CT) or homozygous (TT) and heterozygous (GA) or homozygous (AA), respectively were considered to have an actionable variant in those genes.

## Supporting information

S1 Table Actionable PGx variants identified in the Saudi population. (XLSX)

S2 Table List of rare PGx variants. (XLSX)

S3 Table List of novel PGx variants in the Saudi population. (XLSX)

S4 Table List of genes in the custom PGx gene panel. (XLSX)

S5 Table Classification thresholds and prediction algorithms for novel PGx variants. (XLSX)

## References

[1] Schildcrout JS, Denny JC, Bowton E, Gregg W, Pulley JM, Basford MA, et al. Optimizing drug outcomes through pharmacogenetics: a case for preemptive genotyping. Clin Pharmacol Ther. 2012;92(2):235–42. Epub 2012/06/29. doi: 10.1038/clpt.2012.66 . 22739144 PMC3785311

[2] Johnson JA. Drug target pharmacogenomics: an overview. Am J Pharmacogenomics. 2001;1(4):271–81. Epub 2002/06/27. doi: 10.2165/00129785-200101040-00004 . 12083959

[3] Fujikura K, Ingelman-Sundberg M, Lauschke VM. Genetic variation in the human cytochrome P450 supergene family. Pharmacogenet Genomics. 2015;25(12):584–94. Epub 2015/09/05. doi: 10.1097/FPC.0000000000000172 . 26340336

[4] Kozyra M, Ingelman-Sundberg M, Lauschke VM. Rare genetic variants in cellular transporters, metabolic enzymes, and nuclear receptors can be important determinants of interindividual differences in drug response. Genet Med. 2017;19(1):20–9. Epub 2016/04/22. doi: 10.1038/gim.2016.33 . 27101133

[5] Zhou Y, Ingelman-Sundberg M, Lauschke VM. Worldwide Distribution of Cytochrome P450 Alleles: A Meta-analysis of Population-scale Sequencing Projects. Clin Pharmacol Ther. 2017;102(4):688–700. Epub 2017/04/06. doi: 10.1002/cpt.690 . 28378927 PMC5600063

[6] Caudle KE, Dunnenberger HM, Freimuth RR, Peterson JF, Burlison JD, Whirl-Carrillo M, et al. Standardizing terms for clinical pharmacogenetic test results: consensus terms from the Clinical Pharmacogenetics Implementation Consortium (CPIC). Genet Med. 2017;19(2):215–23. Epub 2016/07/22. doi: 10.1038/gim.2016.87 . 27441996 PMC5253119

[7] Ramos E, Doumatey A, Elkahloun AG, Shriner D, Huang H, Chen G, et al. Pharmacogenomics, ancestry and clinical decision making for global populations. Pharmacogenomics J. 2014;14(3):217–22. Epub 2013/07/10. doi: 10.1038/tpj.2013.24 . 23835662

[8] Johnson JA, Burkley BM, Langaee TY, Clare-Salzler MJ, Klein TE, Altman RB. Implementing personalized medicine: development of a cost-effective customized pharmacogenetics genotyping array. Clin Pharmacol Ther. 2012;92(4):437–9. Epub 2012/08/23. doi: 10.1038/clpt.2012.125 . 22910441 PMC3454443

[9] Ji Y, Skierka JM, Blommel JH, Moore BE, VanCuyk DL, Bruflat JK, et al. Preemptive Pharmacogenomic Testing for Precision Medicine: A Comprehensive Analysis of Five Actionable Pharmacogenomic Genes Using Next-Generation DNA Sequencing and a Customized CYP2D6 Genotyping Cascade. J Mol Diagn. 2016;18(3):438–45. Epub 2016/03/08. doi: 10.1016/j.jmoldx.2016.01.003 . 26947514 PMC4851731

[10] Amstutz U, Henricks LM, Offer SM, Barbarino J, Schellens JHM, Swen JJ, et al. Clinical Pharmacogenetics Implementation Consortium (CPIC) Guideline for Dihydropyrimidine Dehydrogenase Genotype and Fluoropyrimidine Dosing: 2017 Update. Clin Pharmacol Ther. 2018;103(2):210–6. Epub 2017/11/21. doi: 10.1002/cpt.911 . 29152729 PMC5760397

[11] Relling MV, Schwab M, Whirl-Carrillo M, Suarez-Kurtz G, Pui CH, Stein CM, et al. Clinical Pharmacogenetics Implementation Consortium Guideline for Thiopurine Dosing Based on TPMT and NUDT15 Genotypes: 2018 Update. Clin Pharmacol Ther. 2019;105(5):1095–105. Epub 2018/11/18. doi: 10.1002/cpt.1304 . 30447069 PMC6576267

[12] Johnson JA, Caudle KE, Gong L, Whirl-Carrillo M, Stein CM, Scott SA, et al. Clinical Pharmacogenetics Implementation Consortium (CPIC) Guideline for Pharmacogenetics-Guided Warfarin Dosing: 2017 Update. Clin Pharmacol Ther. 2017;102(3):397–404. Epub 2017/02/16. doi: 10.1002/cpt.668 . 28198005 PMC5546947

[13] Birdwell KA, Decker B, Barbarino JM, Peterson JF, Stein CM, Sadee W, et al. Clinical Pharmacogenetics Implementation Consortium (CPIC) Guidelines for CYP3A5 Genotype and Tacrolimus Dosing. Clin Pharmacol Ther. 2015;98(1):19–24. Epub 2015/03/25. doi: 10.1002/cpt.113 . 25801146 PMC4481158

[14] Scott SA, Sangkuhl K, Stein CM, Hulot JS, Mega JL, Roden DM, et al. Clinical Pharmacogenetics Implementation Consortium guidelines for CYP2C19 genotype and clopidogrel therapy: 2013 update. Clin Pharmacol Ther. 2013;94(3):317–23. Epub 2013/05/24. doi: 10.1038/clpt.2013.105 . 23698643 PMC3748366

[15] Van Driest SL, Shi Y, Bowton EA, Schildcrout JS, Peterson JF, Pulley J, et al. Clinically actionable genotypes among 10,000 patients with preemptive pharmacogenomic testing. Clin Pharmacol Ther. 2014;95(4):423–31. Epub 2013/11/21. doi: 10.1038/clpt.2013.229 . 24253661 PMC3961508

[16] Bielinski SJ, Olson JE, Pathak J, Weinshilboum RM, Wang L, Lyke KJ, et al. Preemptive genotyping for personalized medicine: design of the right drug, right dose, right time-using genomic data to individualize treatment protocol. Mayo Clin Proc. 2014;89(1):25–33. Epub 2014/01/07. doi: 10.1016/j.mayocp.2013.10.021 . 24388019 PMC3932754

[17] Relling MV, Klein TE. CPIC: Clinical Pharmacogenetics Implementation Consortium of the Pharmacogenomics Research Network. Clin Pharmacol Ther. 2011;89(3):464–7. Epub 2011/01/29. doi: 10.1038/clpt.2010.279 . 21270786 PMC3098762

[18] Blagec K, Koopmann R, Crommentuijn-van Rhenen M, Holsappel I, van der Wouden CH, Konta L, et al. Implementing pharmacogenomics decision support across seven European countries: The Ubiquitous Pharmacogenomics (U-PGx) project. J Am Med Inform Assoc. 2018;25(7):893–8. Epub 2018/02/15. doi: 10.1093/jamia/ocy005 . 29444243 PMC6016647

[19] Sivadas A, Scaria V. Pharmacogenomic survey of Qatari populations using whole-genome and exome sequences. Pharmacogenomics J. 2018;18(4):590–600. Epub 2018/05/04. doi: 10.1038/s41397-018-0022-8 . 29720721

[20] Wright GEB, Carleton B, Hayden MR, Ross CJD. The global spectrum of protein-coding pharmacogenomic diversity. Pharmacogenomics J. 2018;18(1):187–95. Epub 2016/10/26. doi: 10.1038/tpj.2016.77 . 27779249 PMC5817389

[21] Bush WS, Crosslin DR, Owusu-Obeng A, Wallace J, Almoguera B, Basford MA, et al. Genetic variation among 82 pharmacogenes: The PGRNseq data from the eMERGE network. Clin Pharmacol Ther. 2016;100(2):160–9. Epub 2016/02/10. doi: 10.1002/cpt.350 . 26857349 PMC5010878

[22] Cohn I, Paton TA, Marshall CR, Basran R, Stavropoulos DJ, Ray PN, et al. Genome sequencing as a platform for pharmacogenetic genotyping: a pediatric cohort study. NPJ Genom Med. 2017;2:19. Epub 2017/12/22. doi: 10.1038/s41525-017-0021-8 . 29263831 PMC5677914

[23] Chua EW, Cree SL, Ton KN, Lehnert K, Shepherd P, Helsby N, et al. Cross-Comparison of Exome Analysis, Next-Generation Sequencing of Amplicons, and the iPLEX((R)) ADME PGx Panel for Pharmacogenomic Profiling. Front Pharmacol. 2016;7:1. Epub 2016/02/10. doi: 10.3389/fphar.2016.00001 . 26858644 PMC4726781

[24] Zhou Y, Lauschke VM. Comprehensive overview of the pharmacogenetic diversity in Ashkenazi Jews. J Med Genet. 2018;55(9):617–27. Epub 2018/07/05. doi: 10.1136/jmedgenet-2018-105429 . 29970487

[25] Lek M, Karczewski KJ, Minikel EV, Samocha KE, Banks E, Fennell T, et al. Analysis of protein-coding genetic variation in 60,706 humans. Nature. 2016;536(7616):285–91. Epub 2016/08/19. doi: 10.1038/nature19057 . 27535533 PMC5018207

[26] Caldwell MD, Awad T, Johnson JA, Gage BF, Falkowski M, Gardina P, et al. CYP4F2 genetic variant alters required warfarin dose. Blood. 2008;111(8):4106–12. Epub 2008/02/06. doi: 10.1182/blood-2007-11-122010 . 18250228 PMC2288721

[27] Danese E, Montagnana M, Johnson JA, Rettie AE, Zambon CF, Lubitz SA, et al. Impact of the CYP4F2 p.V433M polymorphism on coumarin dose requirement: systematic review and meta-analysis. Clin Pharmacol Ther. 2012;92(6):746–56. Epub 2012/11/08. doi: 10.1038/clpt.2012.184 . 23132553 PMC3731755

[28] Zhang JE, Jorgensen AL, Alfirevic A, Williamson PR, Toh CH, Park BK, et al. Effects of CYP4F2 genetic polymorphisms and haplotypes on clinical outcomes in patients initiated on warfarin therapy. Pharmacogenet Genomics. 2009;19(10):781–9. Epub 2009/09/11. doi: 10.1097/FPC.0b013e3283311347 . 19741565

[29] Shehab N, Sperling LS, Kegler SR, Budnitz DS. National estimates of emergency department visits for hemorrhage-related adverse events from clopidogrel plus aspirin and from warfarin. Arch Intern Med. 2010;170(21):1926–33. Epub 2010/11/26. doi: 10.1001/archinternmed.2010.407 . 21098354

[30] Kuehl P, Zhang J, Lin Y, Lamba J, Assem M, Schuetz J, et al. Sequence diversity in CYP3A promoters and characterization of the genetic basis of polymorphic CYP3A5 expression. Nat Genet. 2001;27(4):383–91. Epub 2001/03/30. doi: 10.1038/86882 . 11279519

[31] Thompson EE, Kuttab-Boulos H, Witonsky D, Yang L, Roe BA, Di Rienzo A. CYP3A variation and the evolution of salt-sensitivity variants. Am J Hum Genet. 2004;75(6):1059–69. Epub 2004/10/20. doi: 10.1086/426406 . 15492926 PMC1182141

[32] Evans WE, Horner M, Chu YQ, Kalwinsky D, Roberts WM. Altered mercaptopurine metabolism, toxic effects, and dosage requirement in a thiopurine methyltransferase-deficient child with acute lymphocytic leukemia. J Pediatr. 1991;119(6):985–9. Epub 1991/12/01. doi: 10.1016/s0022-3476(05)83063-x . 1960624

[33] Yang JJ, Landier W, Yang W, Liu C, Hageman L, Cheng C, et al. Inherited NUDT15 variant is a genetic determinant of mercaptopurine intolerance in children with acute lymphoblastic leukemia. J Clin Oncol. 2015;33(11):1235–42. Epub 2015/01/28. doi: 10.1200/JCO.2014.59.4671 . 25624441 PMC4375304

[34] Hoff PM, Ansari R, Batist G, Cox J, Kocha W, Kuperminc M, et al. Comparison of oral capecitabine versus intravenous fluorouracil plus leucovorin as first-line treatment in 605 patients with metastatic colorectal cancer: results of a randomized phase III study. J Clin Oncol. 2001;19(8):2282–92. Epub 2001/04/17. doi: 10.1200/JCO.2001.19.8.2282 . 11304782

[35] Del Re M, Quaquarini E, Sottotetti F, Michelucci A, Palumbo R, Simi P, et al. Uncommon dihydropyrimidine dehydrogenase mutations and toxicity by fluoropyrimidines: a lethal case with a new variant. Pharmacogenomics. 2016;17(1):5–9. Epub 2015/12/15. doi: 10.2217/pgs.15.146 . 26651493

[36] Alkuraya FS. Autozygome decoded. Genet Med. 2010;12(12):765–71. Epub 2010/12/30. doi: 10.1097/GIM.0b013e3181fbfcc4 . 21189493

[37] Al-Eitan LN. Pharmacogenomic landscape of VIP genetic variants in Jordanian Arabs and comparison with worldwide populations. Gene. 2020;737:144408. Epub 2020/02/03. doi: 10.1016/j.gene.2020.144408 . 32007583

[38] Ingelman-Sundberg M, Mkrtchian S, Zhou Y, Lauschke VM. Integrating rare genetic variants into pharmacogenetic drug response predictions. Hum Genomics. 2018;12(1):26. Epub 2018/05/26. doi: 10.1186/s40246-018-0157-3 . 29793534 PMC5968569

[39] Abouelhoda M, Sobahy T, El-Kalioby M, Patel N, Shamseldin H, Monies D, et al. Clinical genomics can facilitate countrywide estimation of autosomal recessive disease burden. Genet Med. 2016;18(12):1244–9. Epub 2016/04/29. doi: 10.1038/gim.2016.37 . 27124789

[40] Saudi Mendeliome G. Comprehensive gene panels provide advantages over clinical exome sequencing for Mendelian diseases. Genome Biol. 2015;16:134. Epub 2015/06/27. doi: 10.1186/s13059-015-0693-2 . 26112015 PMC4499193

[41] Browning BL, Zhou Y, Browning SR. A One-Penny Imputed Genome from Next-Generation Reference Panels. Am J Hum Genet. 2018;103(3):338–48. Epub 2018/08/14. doi: 10.1016/j.ajhg.2018.07.015 . 30100085 PMC6128308

[42] Browning SR, Browning BL. Rapid and accurate haplotype phasing and missing-data inference for whole-genome association studies by use of localized haplotype clustering. Am J Hum Genet. 2007;81(5):1084–97. Epub 2007/10/10. doi: 10.1086/521987 . 17924348 PMC2265661

[43] Richards S, Aziz N, Bale S, Bick D, Das S, Gastier-Foster J, et al. Standards and guidelines for the interpretation of sequence variants: a joint consensus recommendation of the American College of Medical Genetics and Genomics and the Association for Molecular Pathology. Genet Med. 2015;17(5):405–24. Epub 2015/03/06. doi: 10.1038/gim.2015.30 . 25741868 PMC4544753

[44] Howe KL, Achuthan P, Allen J, Allen J, Alvarez-Jarreta J, Amode MR, et al. Ensembl 2021. Nucleic Acids Res. 2021;49(D1):D884–D91. Epub 2020/11/03. doi: 10.1093/nar/gkaa942 . 33137190 PMC7778975

[45] Zhou Y, Mkrtchian S, Kumondai M, Hiratsuka M, Lauschke VM. An optimized prediction framework to assess the functional impact of pharmacogenetic variants. Pharmacogenomics J. 2019;19(2):115–26. Epub 2018/09/13. doi: 10.1038/s41397-018-0044-2 . 30206299 PMC6462826

